# 氟功能化锆基金属有机框架负载薄膜微萃取阵列用于草甘膦残留的高通量分析

**DOI:** 10.3724/SP.J.1123.2026.01034

**Published:** 2026-10-08

**Authors:** Sainan LI, Xiaoju YIN, Jiawei LIU, Yang YANG, Quan BAI

**Affiliations:** 1. 合成与天然功能分子教育部重点实验室，陕西省现代分离科学重点实验室，西北大学化学与材料科学学院，陕西 西安 710127; 1. Key Laboratory of Synthetic and Natural Functional Molecule of the Ministry of Education，Key Laboratory of Modern Separation Science in Shaanxi Province，College of Chemistry & Materials Science，Northwest University，Xi’an 710127，China; 2. 核生化灾害防护化学全国重点实验室，北京 102205; 2. State Key Laboratory of Chemistry for NBC Hazards Protection，Beijing 102205，China

**Keywords:** 金属有机框架, 阵列式薄膜微萃取, 草甘膦, 高通量分析, metal-organic frameworks （MOFs）, array-type thin-film microextraction, glyphosate, high-throughput analysis

## Abstract

本研究基于96-刀片式薄膜微萃取（TFME）装置，通过水热法合成氟功能化锆基金属有机框架（UiO-66-F_4_），并与聚丙烯腈（PAN）混合，利用喷涂工艺将其负载于96个不锈钢刀片表面，得到UiO-66-F_4_/PAN涂层阵列。建立的TFME方法可在pH=5~7水溶液中萃取草甘膦，并通过5%（*V*/*V*）三乙胺水溶液实现洗脱。将此方法与UPLC-MS/MS联用，建立了草甘膦高通量分析新方法。该方法线性范围为0.5~500 ng/mL（*R* ^2^=0.999 9），检出限和定量限分别为0.12 ng/mL和0.5 ng/mL，日内（*n*=3）和日间（*n*=5）RSD分别为3.6%和1.6%。该方法成功测得橙子皮和灌溉河水中草甘膦残留量分别为（1.7±0.7） ng/g和（1.2±0.5） ng/mL。样品加标回收率为88.1%~107.1%，精密度（RSD，*n*=3）低于8.1%。该方法准确可靠，重复性好，可并行处理96个样品，适用于大规模样本中草甘膦的快速筛查与准确定量。

## Abstract (en)

The development of high-throughput sample pretreatment methods is essential for the rapid screening and accurate analysis of contaminants in food and environmental matrices. In this study， we established a novel high-throughput sample pretreatment approach for glyphosate using a 96-blade thin-film microextraction （TFME） device. The blade surface was coated with a functionalized metal-organic framework （MOF） as the extractive phase. Specifically， a fluorinated zirconium-based MOF （UiO-66-F_4_） was synthesized *via* a hydrothermal method. Various characterization techniques， including scanning electron microscopy （SEM）， transmission electron microscopy （TEM）， Fourier-transform infrared spectroscopy （FT-IR）， X-ray diffraction （XRD）， and nitrogen adsorption-desorption isotherms， were employed to confirm the successful synthesis of UiO-66-F_4_ crystals. The results demonstrated that the synthesized crystals possessed a high specific surface area （559.5 m^2^/g） and an irregularly stacked porous structure. The prepared UiO-66-F_4_ was subsequently homogenized with a polyacrylonitrile （PAN） solution to form a slurry. This slurry was solidified onto 96 stainless-steel blades using an improved laboratory-developed spraying technique. As a result， an array of 96 UiO-66-F_4_/PAN extraction coatings was obtained. Each coating in the array was fabricated with uniform dimensions of 2 cm in length， 0.2 cm in width， and approximately 100 μm in thickness. The relative standard deviations （RSDs， *n*=8） for the length， width， and thickness of the coatings measured were 0.29%， 0.48%， and 4.64%， respectively. The UiO-66-F_4_ adsorbent offers multiple interaction forces with glyphosate， including metal coordination， hydrogen bonding， and electrostatic interactions. Therefore， the adsorption of glyphosate onto UiO-66-F_4_ occurred rapidly， reaching equilibrium within 5 min. The pseudo-second-order kinetic model provides a superior fit for the adsorption kinetics behavior of glyphosate on the surface of UiO-66-F_4_， suggesting that the adsorption process is predominantly controlled by chemisorption. Furthermore， the glyphosate adsorption isotherms indicate that its adsorption behavior closely conforms to the Langmuir isotherm model （*R* ^2^>0.97）. This finding suggests that glyphosate is adsorbed as a monolayer on the adsorbent surface， giving rise to a maximum adsorption capacity of 239.4 mg/g. The developed TFME method efficiently enriches glyphosate from aqueous solutions at pH 5–7 within 30 min and allows for analyte desorption using an aqueous solution containing 5% （*V*/*V*） triethylamine. The UiO-66-F_4_/PAN coatings displayed high repeatability and reproducibility， as evidenced by the RSD values below 2.3% （*n*=5）. In addition， the coatings exhibited remarkable robustness， maintaining a recovery of 98.2% after nine consecutive TFME processing cycles. Furthermore， coupling this TFME method with ultra performance liquid chromatography-tandem mass spectrometry （UPLC-MS/MS） enabled high-throughput analysis of glyphosate. The method demonstrated excellent linearity over a range of 0.5–500 ng/mL （*R* ^2^=0.999 9）. The limits of detection （LOD） and quantification （LOQ） were 0.12 ng/mL and 0.5 ng/mL， respectively. Intra-day （*n*=3） and inter-day （*n*=5） precisions， expressed as RSDs， were 3.6% and 1.6%， respectively. The method was successfully applied to real-world environmental water and fruit samples， detecting glyphosate residues of （1.7±0.7） ng/g in orange peel and （1.2±0.5） ng/mL in irrigation river water. Spiked recoveries in real samples ranged from 88.1% to 107.1%， with precisions （RSDs， *n*=3） below 8.1%. This method is accurate， reliable， and reproducible， enabling parallel processing of 96 samples， which are well-suited for rapid screening and precise quantification of glyphosate contamination in large-scale sample analyses.

草甘膦是当今世界上使用最多的广谱除草剂之一，通过阻断氨基酸形成的关键途径来抑制有害植物生长^［1］^。经过长期累积及在生态系统中迁移扩散，草甘膦在土壤、地表水、溪流甚至人的体液中均有检出^［2］^。研究表明，接触草甘膦可导致氧化应激、DNA损伤和癌变^［3］^，因而引发人们对土壤、水体和食品中草甘膦残留的关注。草甘膦分子的化学结构中包含膦酸基、仲氨基、羧基等基团，具有强极性和低挥发性^［4，5］^。在实际分析中，草甘膦存在于复杂基质中且含量较低，增加了其检测难度。因此构建高效的样品前处理方法是实现样品中草甘膦准确检测的关键环节。

目前草甘膦残留分析中使用的样品前处理方法有液液萃取^［6］^、QuEChERS^［7］^、固相萃取（SPE）^［8，9］^、分散固相萃取（DSPE）^［10］^和固相微萃取（SPME）^［11］^等，其中液液萃取和固相萃取比较常用。液液萃取主要使用有机溶剂（正己烷、二氯甲烷等）除去样品基质中的非极性杂质，草甘膦保留在水相中^［6，12］^。固相萃取常用商品化的SPE小柱，如阳离子交换柱、C18柱、亲水-亲脂平衡柱（HLB）等净化样品^［12-14］^。例如杨霄等^［14］^使用PRiME HLB小柱对沉积物提取液进行净化，并采用UPLC-MS/MS测定其中的草甘膦、草铵膦及其代谢产物。SPE也可采用新型的分子印迹吸附材料对样品中的草甘膦进行选择性富集^［15，16］^。然而，液液萃取使用有毒有机溶剂，商品化SPE小柱成本较高，且无法重复使用，而分子印迹材料制备过程烦琐耗时，印迹效果不佳，存在较大局限性。此外，现有方法的样品处理通量普遍不足，难以承担大批量样品中草甘膦快速筛查任务。因此，开发一种高通量样品预处理新方法对于草甘膦的高效分析具有重要应用价值。

薄膜微萃取（TFME）是一种由SPME发展而来的微萃取技术^［17，18］^，具有大的萃取相体积和高表面积-体积之比，有利于提高萃取量，缩短萃取平衡时间^［19］^。TFME可设计为多齿几何形状（如96-刀片状），用于负载多个萃取涂层，形成阵列式TFME萃取装置，满足大批量样品的同时处理需求，从而大幅提升样品处理通量，提高分析效率^［20-22］^。因此，TFME技术在样品前处理中具有显著优势，为复杂样品中目标物的高效、高通量分析提供了新的可能。

金属有机框架（MOFs）是通过金属节点与多齿有机配体的定向配位作用而构筑的三维有序多孔框架材料，具有较高的热稳定性和化学稳定性、可调的孔隙大小、可变的框架组成和暴露的活性位点，在样品前处理领域应用广泛^［23-26］^。含氟功能化的锆基MOFs（UiO-66-F_4_）是一种基于UiO-66框架的氟化衍生物，其形成的缺陷结构可以提供更多的活性位点，并且氟原子的引入提高了MOFs的水稳定性和耐酸碱性^［27］^。UiO-66-F_4_可以通过氟原子基团以及锆金属簇与草甘膦分子之间形成较强的氢键、金属配位和静电作用，这些相互作用之间的协同效应能有效增强材料对草甘膦的吸附性能。同时，MOFs的高比表面积和高孔隙率还可以提高对草甘膦的吸附容量。此外，含氟取代基的对苯二甲酸配体易溶于水，故UiO-66-F_4_可在水相中合成，避免了有机试剂的使用，合成过程更加绿色环保^［27］^。

综上所述，本研究采用水热法合成了含氟功能化的MOFs晶体（UiO-66-F_4_），并将其作为制备TFME涂层的材料，以聚丙烯腈（PAN）为黏连剂，通过喷涂法在8支梳状不锈钢支撑体（每支含12齿刀片）表面制备UiO-66-F_4_/PAN萃取涂层，并将其组装成96-刀片状TFME萃取阵列。以草甘膦为分析对象，优化萃取和洗脱参数，建立TFME处理草甘膦的工作流程。进一步结合UPLC-MS/MS检测技术，建立一种用于环境水样和水果样品中草甘膦高通量分析新方法，考察该方法的分析性能，并对橙子和灌溉河水中痕量的草甘膦残留进行分析。

## 1 实验部分

### 1.1 仪器、试剂与材料

ACQUITY UPLC-Xevo TQ-S cronos超高效液相色谱-三重四极杆质谱仪（美国Waters公司）；Mira 4扫描电子显微镜（SEM，捷克TESCAN公司）；JEM-1400透射电子显微镜（TEM，日本Hitachi公司）；TENSOR 27傅里叶变换红外光谱仪（FT-IR）、D8 ADVANCE X射线粉末衍射仪（XRD，德国Bruker公司）；STA 449C型热分析仪（TGA，德国Netzsch公司）；TriStar Ⅱ 3020比表面积和孔隙率分析仪（美国Micromeritics公司）；Concept 96 Kit平板振荡器（德国PAS Technology公司）。

氯化锆（98%）、甲醇（色谱纯）和乙酸铵（≥98.0%）均购自上海阿拉丁试剂有限公司；四氟对苯二甲酸（97%）、草甘膦（95%）、聚丙烯腈（*M*_w_ 150 000）均购自上海麦克林试剂生化科技有限公司；乙腈（色谱纯）购自安徽泽升科技股份有限公司；硫酸（95%~98%）和盐酸（36%~38%）购自四川西陇科学有限公司；*N，N*-二甲基甲酰胺（DMF，99.5%）、氢氧化钠（≥96.0%）、三乙胺（TEA，≥99.0%）、氨水（25%）和甲酸（≥99.5%）均购自天津科密欧化学试剂有限公司；芴甲氧羰酰氯（Fmoc-Cl，≥99.0%）购自上海迈瑞尔生化科技有限公司；四硼酸钠（≥99.5%）购自天津奥普升化工有限公司。实验室用水为超纯水（18 MΩ∙cm，德国默克密理博公司）。

### 1.2 实验方法

#### 1.2.1 UiO-66-F_4_的合成

称取0.47 g氯化锆和0.48 g四氟对苯二甲酸，分别溶于10 mL水中，室温下将二者混合，并加入100 μL硫酸，超声至混合溶液变为乳白色。将混合液转移至50 mL不锈钢反应釜中，90 ℃加热16 h形成UiO-66-F_4_晶体。反应结束后，将产物于5 000 r/min离心3 min，得到白色固体，用水洗涤4次，-65 ℃冷冻干燥24 h。

#### 1.2.2 UiO-66-F_4_/PAN涂层的制备

首先称取0.75 g UiO-66-F_4_固体粉末于6 mL DMF中超声分散均匀，再与4 mL含6%（质量分数）PAN的DMF溶液混合，磁力搅拌6 h形成均匀的匀浆液；其次将含有12齿刀片的梳状不锈钢支撑体（定制刀片总长度4.20 cm，宽度0.20 cm，厚度0.04 cm，结构示意图见附图S1，www.chrom-China.com）依次用砂纸打磨，纯水超声清洗，盐酸刻蚀，乙醇超声清洗，使不锈钢表面变粗糙；随后采用本实验室改进的喷涂方法^［28］^，将上述匀浆液喷涂在裸露的不锈钢基底表面（控制长度为2.0 cm），每次喷涂后迅速将支撑体置于烘箱中100 ℃固化3 min。重复喷涂和固化操作10次，最终得到厚度约100 μm的UiO-66-F_4_/PAN涂层。按照上述步骤共制备96个萃取涂层（8支梳状不锈钢支撑体，每支包含12齿刀片），确保所有涂层的长、宽和厚度保持一致。

#### 1.2.3 实际样品预处理

灌溉河水采集自当地农田附近河流，橙子购自本地农贸市场。河水经过滤除去不溶物后进行分析。橙子样品预先将果皮和果肉剥离，果皮放入榨汁机中打碎为糊状。准确称取2 g果皮样品于50 mL塑料离心管中，加入10 mL水和10 mL二氯甲烷，振荡提取20 min，于4 000 r/min离心10 min，收集上层水相，残渣再加入10 mL水重复提取一次，合并上层水相。果肉处理步骤与果皮相同。

#### 1.2.4 TFME工作流程与样品衍生

将含有96个UiO-66-F_4_/PAN涂层的萃取阵列安装在Concept 96 Kit平板振荡器上，溶液置于96深孔塑料板（2.0 mL）中。TFME流程包括涂层活化、萃取、清洗和洗脱步骤。首先，向深孔板各个孔内加入1.5 mL甲醇/水（1:1，*V*/*V*）溶液，以500 r/min的速率振荡10 min以活化涂层；其次向各个孔内分别加入1 mL样品溶液（pH 5~7），以500 r/min的速率振荡萃取30 min；随后将涂层浸没在纯水中并振荡10 s，对涂层表面进行清洗；最后用1 mL 5%（*V*/*V*）三乙胺水溶液以500 r/min的速率振荡洗脱2 h。

草甘膦分析前进行衍生处理。衍生前取0.5 mL洗脱液，在65 ℃下通过氮气吹扫除尽溶剂，再加入0.5 mL纯水复溶。衍生过程：向复溶样品溶液中加入200 μL四硼酸钠水溶液（26 mg/mL），涡旋1 min，再加入300 μL衍生试剂Fmoc-Cl乙腈溶液（1.0 mg/mL），涡旋混匀1 min，室温下过夜反应。衍生液经0.22 μm微孔滤膜过滤，通过UPLC-MS/MS分析。

#### 1.2.5 UPLC-MS/MS分析条件

Shim-pack GISS-HP C18分析柱（150 mm×2.1 mm，3 μm）；流速0.3 mL/min；柱温40 ℃；进样体积5 μL。流动相A为5 mmol/L乙酸铵水溶液，流动相B为乙腈。梯度洗脱程序：0~3.5 min，10%B~33%B；3.5~6 min，33%B~50%B；6~7 min，50%B~80%B；7~7.5 min，80%B~10%B；7.5~9 min，10%B。

电喷雾离子源（ESI）正离子扫描模式，喷雾电压：3 000 V；离子源温度：150 ℃；去溶剂气流量：15 L/min；去溶剂气温度：500 ℃；采用多反应离子监测模式（MRM）进行定量分析。草甘膦的定量离子对为*m*/*z* 392.0>88.0，碰撞能量为25 eV；定性离子对为*m*/*z* 392.0>214.0，碰撞能量为10 eV。

## 2 结果与讨论

### 2.1 UiO-66-F_4_晶体和UiO-66-F_4_/PAN涂层的表征

本实验通过SEM和TEM观察UiO-66-F_4_晶体的表面形貌和内部结构以及UiO-66-F_4_/PAN萃取涂层的表面形貌，结果如图1所示。由图1a可知，水热合成的UiO-66-F_4_晶体粒径较小，且发生明显团聚形成不规则块状形貌。同样在TEM图中观察到UiO-66-F_4_晶体颗粒呈现堆积状且颗粒之间形成孔隙结构（图1b）。由图1c和1d可知，不锈钢基体表面牢固覆盖一定厚度的UiO-66-F_4_/PAN涂层，呈现粗糙状态，并且UiO-66-F_4_晶体分布在涂层中，与PAN聚合物形成共混物。

**图1:**
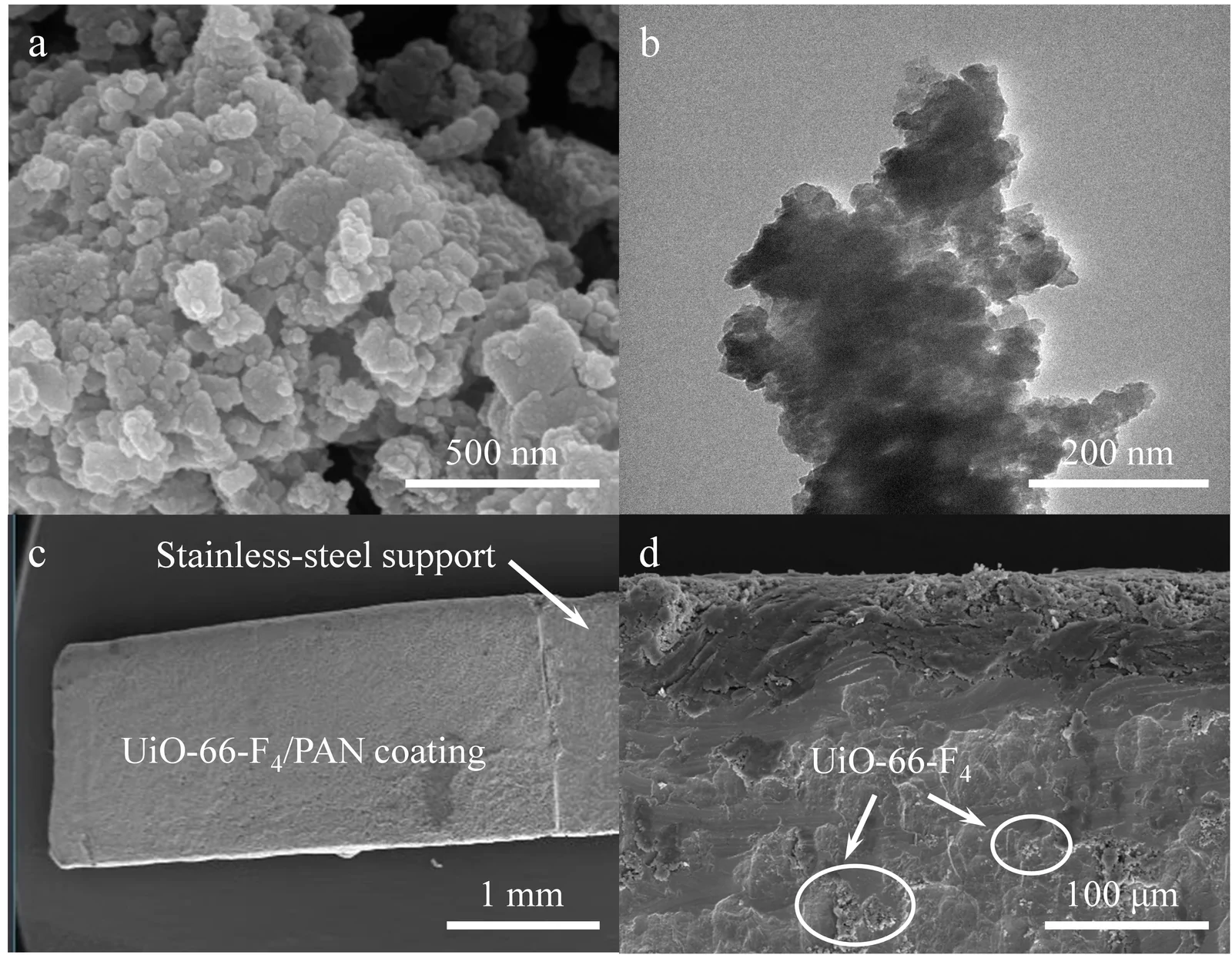
合成的UiO-66-F_4_的（a）扫描电子显微镜照片和（b）透射电子显微镜照片；制备的UiO-66-F_4_/PAN涂层的（c、d）扫描电子显微镜图片

图2a为UiO-66-F_4_和UiO-66-F_4_/PAN的红外光谱图。二者在998、1 270和1 620 cm^-1^处均存在配体C-F及C=O键伸缩振动和弯曲振动引起的红外吸收峰，并且在3 430和660 cm^-1^处的吸收峰属于O-H和Zr-O的伸缩振动。此外，UiO-66-F_4_/PAN在2 242和2 930 cm^-1^处的吸收峰属于PAN聚合物中-CN和-CH_2_的伸缩振动特征峰，证明UiO-66-F_4_与PAN实现了有效混合。XRD表征进一步证实了UiO-66-F_4_和UiO-66-F_4_/PAN的形成（图2b），其中UiO-66-F_4_与UiO-66晶体均在7.4°、8.5°和25.8°处有明显的衍射峰，且所有衍射峰位置基本一致，证明二者具有相同的晶体结构，氟原子取代基团并未影响配体与Zr金属簇之间的连结与组装。PAN粉末在17°处有一衍射峰，但在UiO-66-F_4_/PAN中该衍射峰消失，表明PAN聚合物分子链在溶解前后发生了从有序态向无序态的转变。在UiO-66-F_4_/PAN中同样观察到属于UiO-66-F_4_的特征衍射峰，进一步证实UiO-66-F_4_与PAN之间的物理混合作用。

**图2:**
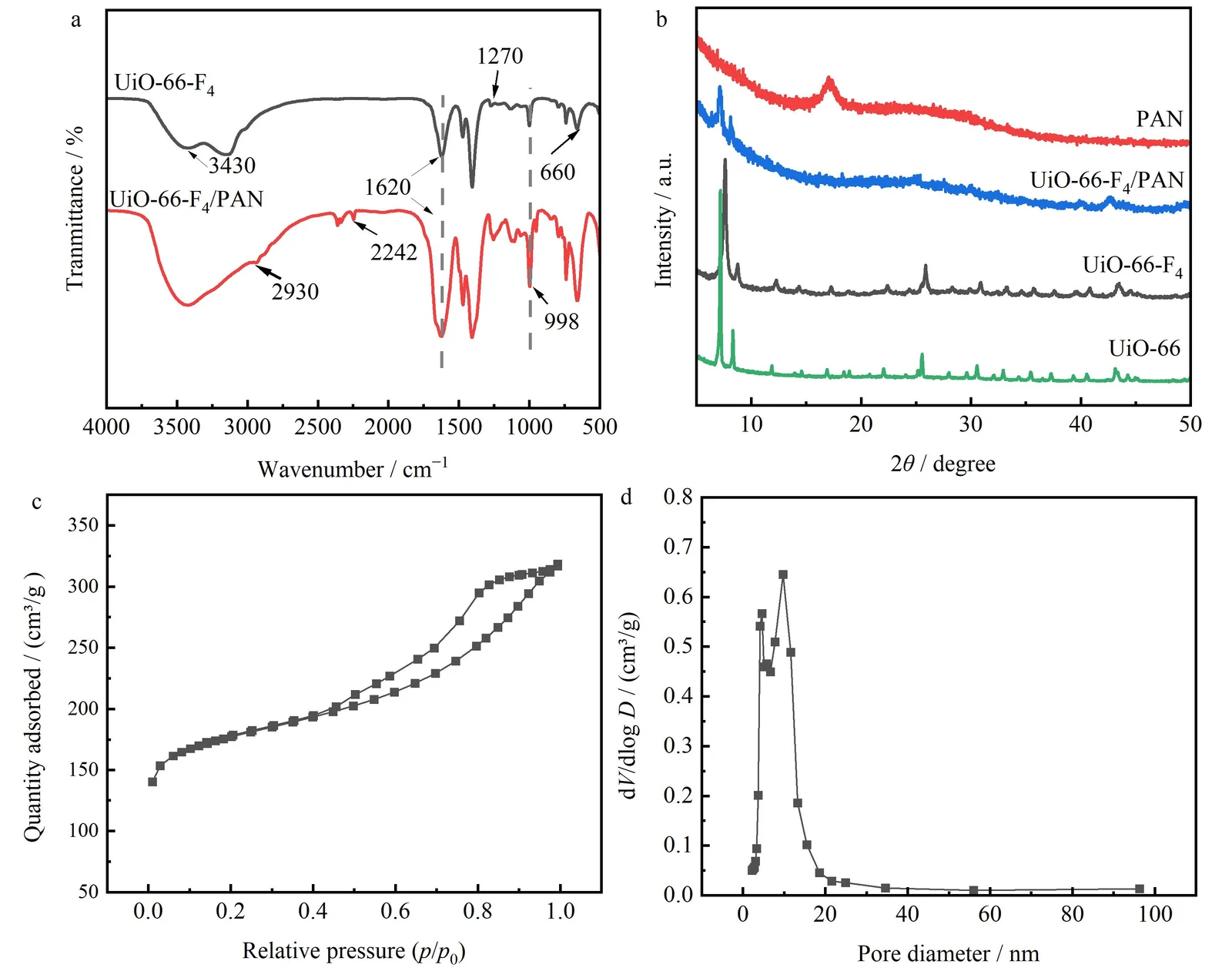
UiO-66-F_4_和UiO-66-F_4_/PAN涂层的（a）红外光谱图和（b）X-射线粉末衍射图；UiO-66-F_4_的（c）氮气吸附-脱附等温曲线图和（d）孔径分布曲线图

本实验通过N_2_等温吸附-脱附实验测定了UiO-66-F_4_的孔隙结构。如图2c所示，UiO-66-F_4_的等温吸附-脱附曲线符合Ⅳ型等温线分类标准，回滞环出现在相对压力较高区间（*p*/*p*_0_=0.7~1.0），表明UiO-66-F_4_存在介孔结构。孔径分布曲线（图2d）表明，UiO-66-F_4_中存在两种不同的介孔结构（平均孔径为4.58和9.69 nm）。该介孔结构可认为是MOF晶体颗粒不规则堆积所产生的孔隙。同时，UiO-66-F_4_具有较高的比表面积（559.5 m^2^/g），可为草甘膦的吸附提供更多的结合位点，并且介孔孔隙能够提高目标物在材料内部的传质效率。UiO-66-F_4_和UiO-66-F_4_/PAN的热重分析（TGA）结果见附图S2。其中UiO-66-F_4_的主要失重温度范围为<150 ℃和350~550 ℃，分别对应MOFs晶体表面或内部残余水分子的挥发以及有机配体的高温热分解。而UiO-66-F_4_/PAN的失重发生在100~300 ℃和300~600 ℃两个升温阶段，前者主要是水和DMF残留溶剂的挥发，后者则包含了PAN聚合物骨架及MOFs有机配体热分解两个部分。TGA测试结果进一步证明了UiO-66-F_4_与PAN的成功混合。

### 2.2 UiO-66-F_4_对草甘膦的吸附性能

本实验考察了UiO-66-F_4_作为吸附剂，在室温下对草甘膦的吸附动力学曲线和吸附等温线。如图3a所示，草甘膦在材料表面具有较快的吸附速率，在5 min时达到吸附平衡。这与UiO-66-F_4_存在介孔结构，能够加快草甘膦传质速率有关，而且吸附过程中，吸附剂分散在草甘膦水溶液中，其表面与草甘膦分子之间的接触几率高，也大大提高了吸附速率。经动力学模型拟合后发现，准二级动力学模型更符合草甘膦在UiO-66-F_4_表面的吸附行为，说明该吸附过程由化学吸附主导，即草甘膦分子与吸附剂之间的氢键作用（氟原子基团提供）、配位作用（Zr金属簇与膦酸基）以及静电作用（金属离子与羧酸根离子）等相互作用力促进了其在UiO-66-F_4_表面的吸附。进一步考察草甘膦的吸附等温线发现（图3b），其平衡吸附容量与平衡浓度之间符合Langmuir等温模型（*R* ^2^>0.97），表明草甘膦在吸附剂表面发生单层吸附，最大静态吸附容量为239.4 mg/g。因此UiO-66-F_4_具有优异的吸附容量和快速吸附特征，适合作为TFME涂层材料用于草甘膦的萃取。

**图3:**
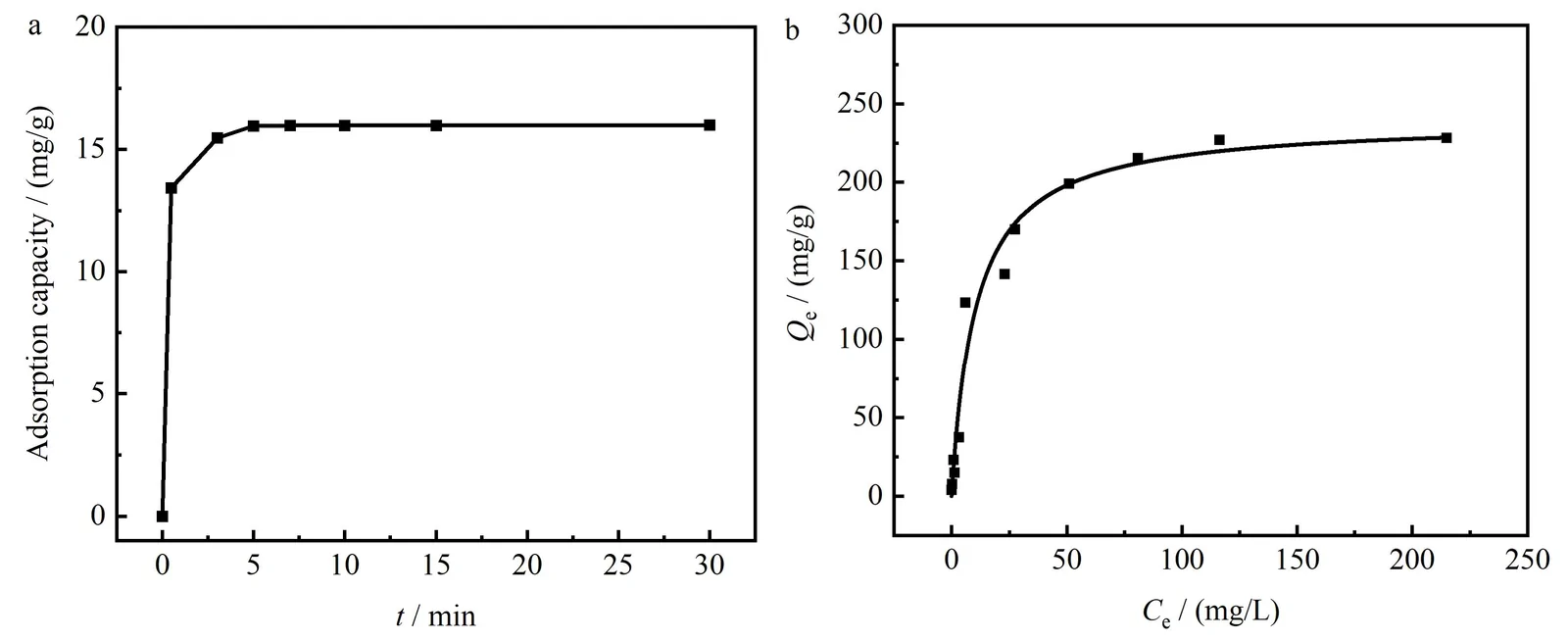
（a）UiO-66-F_4_对草甘膦的吸附时间曲线和（b）Langmuir模型拟合吸附等温线 *Q*_e_： adsorption capacity at adsorption equilibrium； *C*_e_： glyphosate concentration in the supernatant at adsorption equilibrium.

### 2.3 UiO-66-F_4_/PAN涂层制备的优化

为了获得稳定耐用的萃取涂层，本实验对喷涂法制备TFME涂层的参数进行了系统优化，包括UiO-66-F_4_材料用量（0.25、0.5、0.75 g），PAN质量分数（4%、6%、8%），固化温度（80、100、150 ℃）和喷涂次数（5、10、15次），采用单因素变量法，以涂层的稳定性和均匀性以及刀片表面涂层覆盖程度等作为评价指标，最终确定的最优参数如下：将0.75 g吸附剂加入含6% PAN的DMF溶液中，喷涂10次，每次固化温度为100 ℃。不同参数制备的UiO-66-F_4_/PAN涂层见附图S3和附图S4。在此最优条件下，一共在8支梳状不锈钢支撑体上制备了96个UiO-66-F_4_/PAN涂层（图4a），并且所有涂层保持长度2.0 cm，宽度0.2 cm，厚度约100 μm。随机选取8个UiO-66-F_4_/PAN涂层测量，涂层长度、宽度和厚度尺寸的RSD分别为0.29%、0.48%和4.64%，证明了涂层尺寸的一致性，为后续草甘膦的准确分析提供了可靠保障。最后将8支含有涂层的不锈钢支撑体整合安装到平板振荡器上，形成阵列式TFME萃取装置，可同时完成96个样品的预处理操作（图4b）。

**图4:**
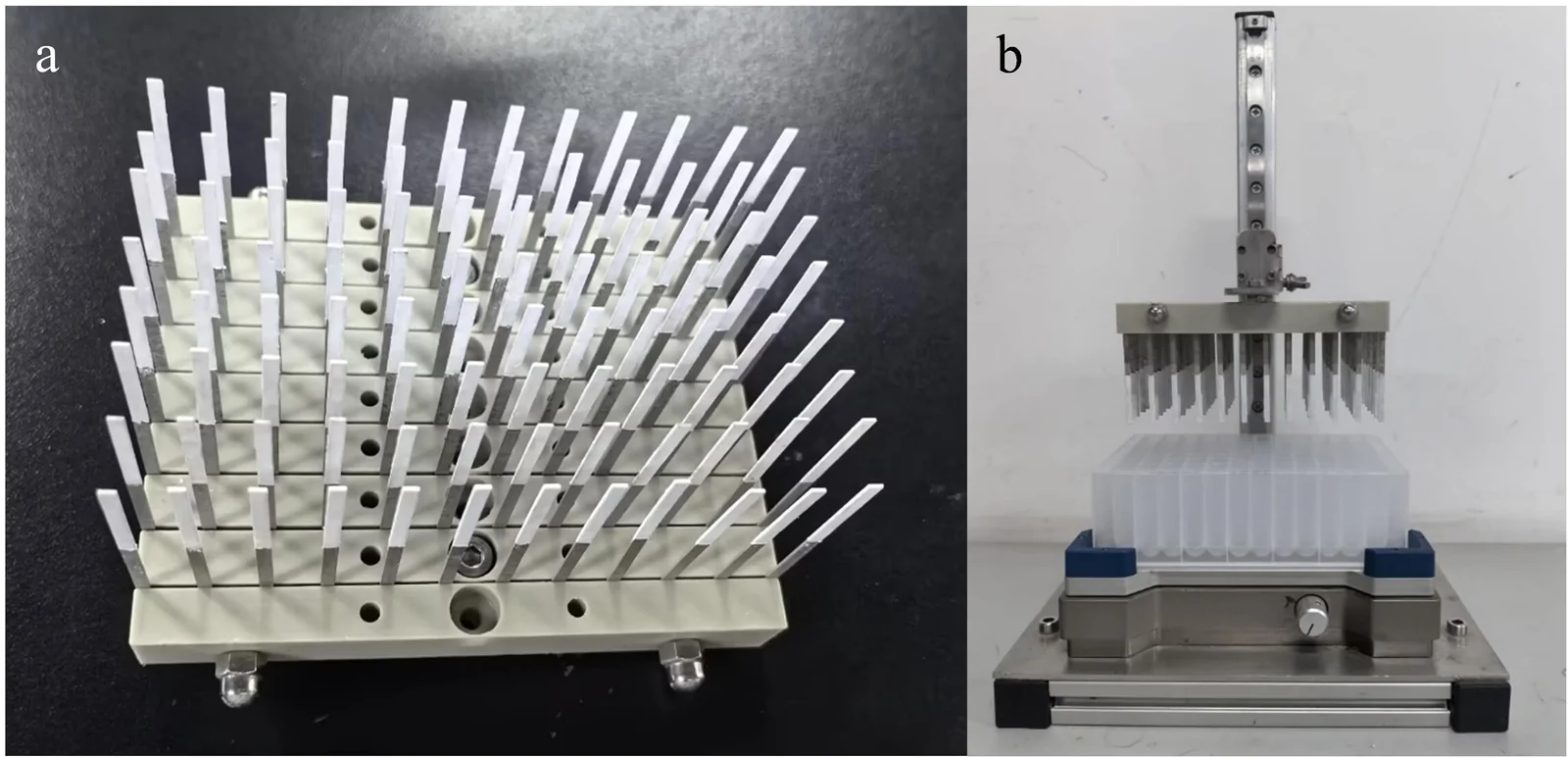
（a）96个UiO-66-F_4_/PAN涂层组装的薄膜微萃取阵列；（b）半自动高通量萃取装置

### 2.4 TFME工作流程的优化

为了达到最佳的萃取效果，采用单因素变量法对影响TFME工作流程中萃取和洗脱两个步骤的因素开展条件实验，以获得最优条件。

#### 2.4.1 pH与盐含量

本实验首先考察了UiO-66-F_4_/PAN涂层在pH 0.5~11范围内对草甘膦萃取的影响。如图5a所示，该涂层在酸性和中性条件下对草甘膦的萃取效果明显优于碱性条件。在中低pH条件下（pH≤7），草甘膦的膦酸基团（-PO_3_H_2_）与UiO-66-F_4_的Zr金属节点会产生较强的配位作用和静电作用^［29］^，同时，氟原子取代基还可以与草甘膦的仲氨基之间形成氢键。但碱性条件下，由于OH^－^的竞争作用，Zr金属节点与膦酸基团之间的配位作用被严重削弱^［30］^，导致萃取量显著下降。进一步考察样品溶液中盐含量对萃取的影响，结果表明（图5b），在含有0~50 mg NaCl范围的样品溶液中，随着盐含量的增加，UiO-66-F_4_/PAN涂层对草甘膦的萃取量逐渐下降。这是因为在高盐浓度下，草甘膦与涂层之间的静电引力被削弱；同时大量阴离子可能会占据Zr-O簇开放的金属位点，一定程度上会阻碍草甘膦与Zr金属节点的配位作用，导致萃取量减少。因此，在进行草甘膦萃取时，样品溶液的pH应控制在5~7范围内，且不添加任何盐。

**图5:**
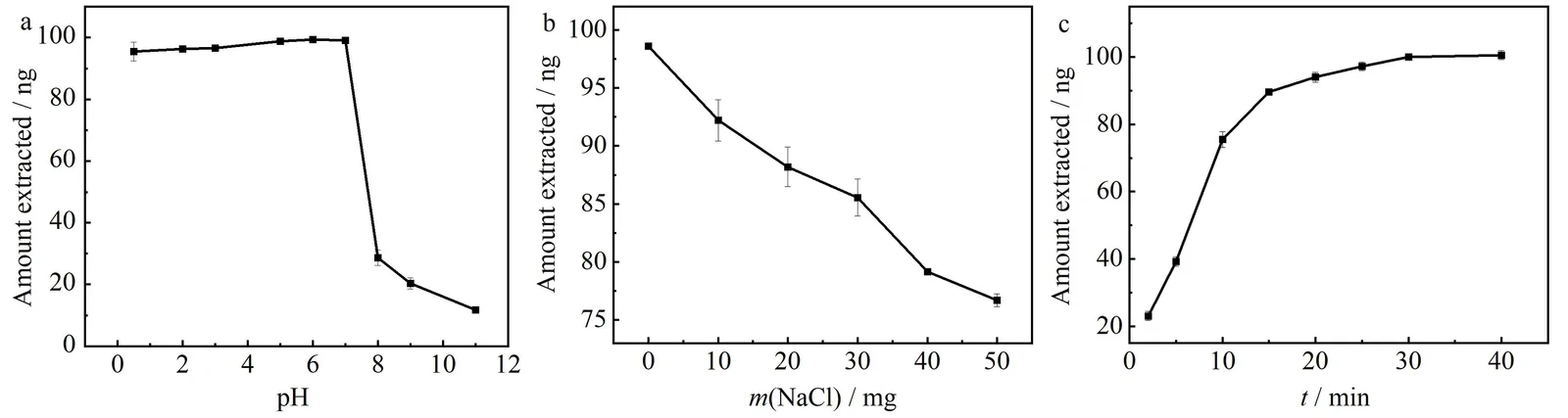
（a）样品溶液pH、（b）样品溶液盐含量及（c）萃取时间对草甘膦萃取性能的影响（*n*=3）

#### 2.4.2 平衡时间

TFME与SPME原理相同，都是依据萃取达到平衡后，涂层中的萃取量*n*与样品中目标物的初始质量浓度*C*_0_之间的定量关系进行分析^［19］^，因此优化萃取平衡时间，保证萃取量十分重要。本实验考察了2~40 min内，UiO-66-F_4_/PAN涂层对草甘膦的萃取动力学曲线（图5c），结果表明，萃取量随时间延长而增加，并在30 min左右达到平衡。相较于UiO-66-F_4_吸附剂对草甘膦萃取的快速平衡结果（5 min），涂层中因为加入PAN作为黏连剂，导致UiO-66-F_4_表面包覆了聚合物层，草甘膦分子需首先穿透该聚合物层后才能进入材料内部，从而延长了萃取时间。此外，由于PAN的固定化作用，限制了UiO-66-F_4_在涂层中的移动，造成材料与草甘膦分子的接触几率减小，使达到萃取平衡的时间延后。尽管如此，PAN作为黏连剂具有抗生物污染的功能^［31］^，可以有效避免样品基质中干扰组分的非特异性吸附，提高净化效果。

#### 2.4.3 洗脱剂类型

草甘膦易溶于水，不溶于一般有机溶剂^［32］^，因此洗脱剂需以水作为溶剂。根据草甘膦与UiO-66-F_4_之间的相互作用机制，洗脱时需在水溶液中加入一定量的碱性物质，从而削弱/破坏二者之间的氢键、配位和静电作用力。图6a为不同洗脱剂对草甘膦的洗脱效果，相较于5%（*V*/*V*）氨水溶液，5%（*V*/*V*）三乙胺水溶液的洗脱效果更佳，而且随着三乙胺含量的增加，草甘膦的洗脱量逐渐增加。这是因为三乙胺作为胺类有机碱，其碱性高于同浓度的氨水，并且随着三乙胺含量的升高，洗脱液碱性增强，对草甘膦与涂层之间的相互作用力削弱能力更强，故洗脱效果增强。但三乙胺含量过高会造成洗脱量下降。此外，涂层在氨水溶液中浸泡后会发生开裂现象，但在三乙胺水溶液中保持完好。综上，本实验选择5%（*V*/*V*）三乙胺水溶液进行洗脱。

**图6:**
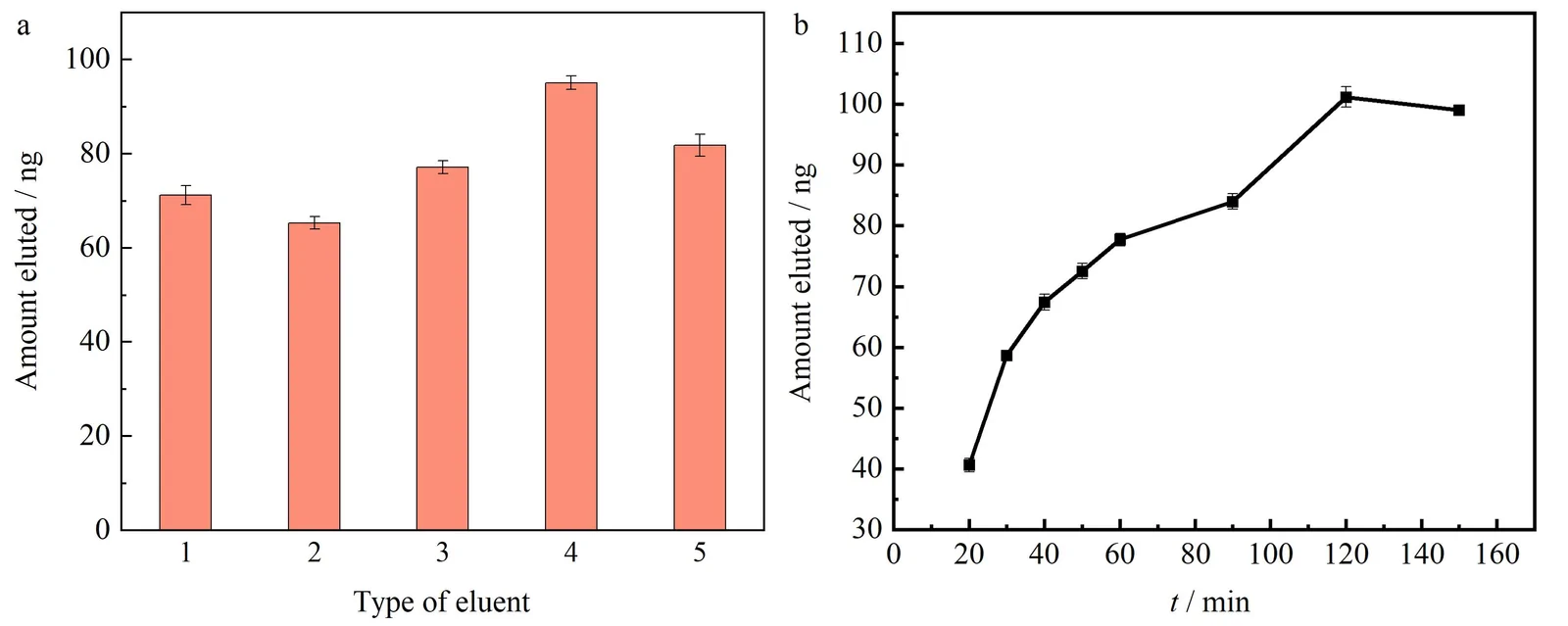
（a）洗脱剂类型及（b）洗脱时间对草甘膦洗脱性能的影响（*n*=3）

#### 2.4.4 洗脱时间

为了达到最佳洗脱效果，实验进一步优化了洗脱时间。如图6b所示，随着洗脱时间增加，洗脱量逐渐增加，在120 min左右达到洗脱量最大值。较长的洗脱时间可能与草甘膦和UiO-66-F_4_/PAN涂层之间的较强相互作用力有关，造成洗脱比较困难。然而借助本实验所用的含96个萃取涂层的TFME阵列，可以在同一时间内进行多个样品的同步洗脱，一定程度上抵消因洗脱时间长而产生的不利影响。

### 2.5 分析方法的建立与评价

在最优工作流程下，本实验将开发的TFME方法与UPLC-MS/MS分析技术相结合，建立了一种高效、高通量分析新方法用于实际样品中草甘膦的分析检测。系统考察了该方法的线性范围及相关系数（*R* ^2^）、检出限（LOD）、定量限（LOQ）和日内及日间精密度（RSD）。草甘膦在0.5~500 ng/mL范围内具有良好的线性关系（*R* ^2^=0.999 9），根据信噪比标准计算该方法的LOD（*S*/*N*=3）和LOQ（*S*/*N*=10）分别为0.12 ng/mL和0.5 ng/mL。实验在100 ng/mL水平下对该分析方法的日内和日间精密度进行了考察，日内测定早、中、晚3个时间段的平行样品，日间连续测定5天，得到日内（*n*=3）和日间（*n*=5）RSD分别为3.6%和1.6%，证明本实验建立的新方法具有良好的重复性和可靠性。

本实验进一步考察了UiO-66-F_4_/PAN涂层的重复使用性和批次制备的重复性。在最佳条件下，使用同一涂层对100 ng/mL水平的草甘膦标准溶液连续完成9次TFME工作流程，测得9次草甘膦平均回收率为98.2%，RSD为2.8%，证明该涂层具有出色的重复使用性能，能够多次进行样品分析。实验进一步考察了多个批次（*n*=5）制备的UiO-66-F_4_/PAN涂层的萃取性能，结果证明草甘膦在不同批次制备的涂层上的回收率为90.2%~102.2%，RSD为2.3%，展现了该涂层良好的制备重复性。上述结果证明本实验开发的UiO-66-F_4_/PAN涂层具有较高的分析可靠性和涂层耐用性，能够保证样品中草甘膦分析结果的准确性。

### 2.6 与其他方法的对比

表1对比了本研究建立的草甘膦高通量分析方法与已有文献报道方法的性能。根据方法的分析性能和单个样品处理时间两个方面的比较结果可知，本方法在回收率与精密度上与现有方法相当，表明其测定结果具有良好的准确性与可靠性。在线性范围和检出限方面，本方法与已报道方法持平或更优，体现出较高的检测灵敏度，能够满足实际样品中痕量草甘膦残留的定量检测需求。尤为突出的是，本方法在分析通量上具备显著优势。尽管样品前处理的总耗时为160 min，但借助96阵列式TFME装置，可实现96个样品同步并行处理，折算后单个样品平均处理时间仅约1.7 min，大幅提升了整体分析效率，可为大批量样品中草甘膦的快速筛查与高通量检测提供技术支撑。

**表1:** 本方法与其他已报道方法的比较

Sample matrix	Method	Adsorptive phase	Analytical performances	Pretreatment time	Ref.
Commercial baby foods	DSPE-CE-MS	CSMS@TiO_2_	linear range： 2‒1 000 ng/mL， LOD： 0.5 ng/mL， recovery： 82.3%‒102.6%， RSD： 2.1%‒8.3%	50 min	［^10^］
Sediment	SPE-UPLC-MS/MS	PRiME HLB column	linear range： 2.0‒200 ng/mL， LOD： 10 ng/g， recovery： 76.4%‒88.8%， RSD： 3.92%‒8.20%	NA	［^14^］
Water， soil and vegetable	MSPE-HPLC	MIH-MIP	linear range： 72‒2 000 ng/mL， LOD： 21 ng/mL， recovery： 88%‒118%， RSD： 2%‒9%	2.3 min	［^15^］
Tea	SPE-LC-MS/MS	DMIP	linear range： 50‒4 000 ng/mL， LOD： 2.8 ng/mL， recovery： 98.69%‒106.26%， RSD： 0.91%‒1.81%	NA	［^16^］
Environmental water and honey	MIP-SPE-HPLC	AFFINIMIP^®^ SPE cartridge	linear range： 0.25‒5 ng/mL， LOD： 0.31 ng/mL， recovery： 97.6%‒103%， RSD： 0.29%‒6.78%	30 min	［^33^］
Environmental water	MSPE-UPLC-MS	PSA@Zr-MOF@Fe_3_O_4_	linear range： 1.0‒100 ng/mL， LOD： 0.02 ng/mL， recovery： 86.3%‒92.1%， RSD： 1.2%‒7.2%	10 min	［^34^］
Plant commodities	Online SPE-LC-MS/MS	ZrO_2_	linear range： 2‒2 000 ng/mL， LOD： NA， recovery： 80%‒120%， RSD： 0.8%‒12.8%	35 min	［^35^］
River water and orange	TFME-UPLC-MS/MS	UiO-66-F_4_	linear range： 0.5‒500 ng/mL， LOD： 0.12 ng/mL， recovery： 88.1%‒107.1%， RSD： 0.7%‒8.1%	160 min for 96 sample， 1.7 min for per sample	this work

Pretreatment time includes required times for pre-condition， extraction and elution of per sample. NA represents the data is not mentioned. CSMS@TiO_2_： titanium dioxide-coated core-shell silica microspheres； MIH-MIP： mixed ion hydroxide molecularly imprinted polymer； DMIP： dummy molecularly imprinted material； PSA@Zr-MOF@Fe_3_O_4_： *N*-（*n*-propyl）ethylenediamine modified magnetic metal-organic framework composite.

### 2.7 实际样品分析

本实验以橙子和灌溉河水两类样本为例，验证所建立的TFME-UPLC-MS/MS分析方法对实际样品中草甘膦检测的实用性。如表2所示，在橙子皮和灌溉河水中检测出痕量的草甘膦残留，含量分别为（1.7±0.7） ng/g和（1.2±0.5） ng/mL。为进一步验证方法的准确度和精密度，橙子果皮和果肉样品分别在25、250和2 000 ng/g 3个水平下进行实际样品的加标试验，测得加标回收率为88.1%~101.4%，RSD低于8.1%（*n*=3）。灌溉河水样品分别在5、50和400 ng/mL 3个水平下进行加标试验，测得加标回收率为96.1%~107.1%，RSD低于2.5%（*n*=3）。加标回收试验结果证明，所建立的草甘膦分析方法具有良好的准确度和精密度，适用于水果和河水样品中草甘膦残留的准确检测。

**表2:** 橙子和灌溉河水中草甘膦的检测结果和3个水平下的加标回收率及精密度（*n*=3）

Sample	Found	Low		Medium		High	
		Recovery/%	RSD/%	Recovery/%	RSD/%	Recovery/%	RSD/%
Orange pulp	N.D.	101.4	3.1	92.5	5.0	90.5	2.7
Orange peel	（1.7±0.7） ng/g	88.1	5.4	95.7	8.1	98.3	4.5
River water	（1.2±0.5） ng/mL	96.1	2.1	107.1	2.5	101.6	0.7

Low， medium， and high levels： for orange samples， 25， 250， and 2 000 ng/g； for river water samples， 5， 50， and 400 ng/mL. N.D.： not detected.

## 3 结论

本研究采用水热法合成了含氟功能化MOFs材料（UiO-66-F_4_）并作为TFME萃取材料，通过实验室改进的喷涂技术将其固定在多齿不锈钢刀片表面，制备了96个尺寸均一、牢固稳定的UiO-66-F_4_/PAN涂层，并将其组装形成高通量TFME萃取阵列。UiO-66-F_4_因与草甘膦分子之间的多重相互作用以及MOFs材料高比表面积和介孔结构，对草甘膦表现出良好的萃取性能。将开发的TFME方法与UPLC-MS/MS检测技术结合，建立了复杂样品中草甘膦的高通量分析新方法，成功应用于水果和环境水样基质中草甘膦残留的检测。此外，含有96个萃取涂层的TFME阵列装置可以同步对96个样品进行前处理操作，大幅提高了分析效率，为大规模样品中草甘膦的高通量筛查分析提供了新的技术支持。
